# Deep phenotyping reveals CRH and FKBP51-dependent behavioral profiles following chronic social stress exposure in male mice

**DOI:** 10.1038/s41386-024-02008-9

**Published:** 2024-10-22

**Authors:** Veronika Kovarova, Joeri Bordes, Shiladitya Mitra, Sowmya Narayan, Margherita Springer, Lea Maria Brix, Jan M. Deussing, Mathias V. Schmidt

**Affiliations:** 1 Research Group Neurobiology of Stress Resilience, Max Planck Institute of Psychiatry, Munich, Germany; 2 International Max Planck Research School for Translational Psychiatry (IMPRS-TP), 80804 Munich, Germany; 3 Research Group Molecular Neurogenetics, Max Planck Institute of Psychiatry, Munich, Germany

**Keywords:** Limbic system, Stress and resilience

## Abstract

The co-chaperone FKBP51, encoded by *FKBP5* gene, is recognized as a psychiatric risk factor for anxiety and depressive disorders due to its crucial role in the stress response. Another key modulator in stress response regulation is the corticotropin releasing hormone (CRH), which is co-expressed with FKBP51 in many stress-relevant brain-regions and cell-types. Together, they intricately influence the balance of the hypothalamic-pituitary-adrenal (HPA) axis, one of the primary stress response systems. Previous research underscores the potential moderating effects these genes have on the regulation of the stressful life events towards the vulnerability of major depressive disorder (MDD). However, the specific function of FKBP51 in CRH-expressing neurons remains largely unexplored. Here, through deep behavioral phenotyping, we reveal heightened stress effects in mice lacking FKBP51 in CRH co-expressing neurons (*CRH*^*FKBP5−/−*^), particularly evident in social contexts. Our findings highlight the importance of considering cell-type specificity and context in comprehending stress responses and advocate for the utilization of machine-learning-driven phenotyping of mouse models. By elucidating these intricacies, we lay down the groundwork for personalized interventions aimed at enhancing stress resilience and individual well-being.

## Introduction

Stress-related disorders constitute a substantial societal burden, impacting both mental and physical health [1, 2]. Defined as a state of threat to bodily homeostasis caused by extrinsic or intrinsic challenges, stress in its social form pervades our social fabric as the most prevalent form in vertebrates, shaping individual and communal behavior [3, 4]. Consequently, it looms as a well-established risk factor for a range of psychiatric diseases, including anxiety disorder, major depressive disorder (MDD), post-traumatic stress disorder (PTSD) [5, 6].

Animal models, like chronic social defeat stress (CSDS), have been instrumental in dissecting the molecular, functional, and behavioral consequences of social stress [7, 8]. Reductionistic approaches at the behavioral level have examined the impact of chronic stress on social dominance and avoidance [9]. Recent advances in computer vision and machine learning-based analysis tools has reached new depths, offering a nuanced understanding of individual responses to stress beyond traditional read-outs [10–19]. Despite these advances, the molecular underpinnings driving these overt differences remain incompletely understood.

The neuroendocrine hallmark of chronic stress is the dysregulation of the hypothalamic-pituitary-adrenal (HPA) axis, a key player in stress-induced psychiatric conditions like depression [20]. For example, rodents with a history of social stressors often exhibit a blunted cortisol response, leading to persistent HPA axis dysregulation [20]. The glucocorticoid (GR) receptor serves as a central regulator of glucocorticoid (cortisol in humans, corticosterone in most rodents) feedback. Acting predominantly as a transcription factor, cytosolic GR is bound to a number of chaperones and co-chaperones, which regulate its ligand sensitivity and nuclear translocation. The co-chaperone FKBP51, encoded by the *FKBP5* gene, is playing a pivotal role in this process [21].

*FKBP5* has consistently been linked to stress-related disorders in humans, with elevated levels correlating with amplified stress sensitivity and greater susceptibility to depression [22, 23]. Acting as a co-chaperone of GR, it modulates the cortisol-receptor complex transport and regulates gene expression, impacting individuals’ stress response [5, 24, 25]. The mechanisms governing GR binding and trafficking thereby finely tune the stress response [20]. Environmental stressors as well as corticosterone treatment intricately regulate the *FKBP5* gene, leading to alterations in methylation and gene expression across tissues [26–28]. Recent findings highlight the profound cell-type and region specific impact of FKBP51 brain expression on acute stress responsiveness, memory, and anxiety behavior in mice [21, 29–31].

Another key stress response modulator is the neuropeptide corticotropin-releasing hormone (CRH) [32, 33]. Abundantly expressed in the hypothalamic paraventricular nucleus (PVN) neurons, CRH assumes a pivotal role in HPA axis regulation and exhibits multifaceted functions across brain regions and neuronal types [34, 35]. Its influence extends to complex behavioural phenotypes, affecting behaviors linked to environmental assessment, vigilance, risk avoidance, and self-directed actions [36].

Both FKBP51 and CRH are co-expressed in numerous stress-relevant regions and cell-types, intricately influencing HPA axis responses [23]. Genetic variations in *FKBP5* and *CRHR1*, one of the receptors of CRH, have been associated with various neuropsychiatric disorders, suggesting a strong genetic aetiology for cortisol response to stress [37]. Meta-analyses highlight the potential moderating effects of these genes (FKBP5, CRH, CRHR1) on HPA axis regulation and the impact of stressful life events on depression, with FKBP5 being particularly notable [38]. However, the detailed function of FKBP51 in CRH-expressing neurons remains elusive.

To address this question, we generated a mouse line lacking FKBP51 expression in CRH-expressing neurons and characterized its phenotype in response to social stress. Leveraging automated behavior phenotyping techniques, we discerned distinct stress- and context-dependent behavioral changes, providing insights into factors influencing vulnerability or resilience to social stressors.

## Materials and Methods

### Animals and animal housing

All protocols and experiments were performed in accordance with the European Communities’ Council directive 2010/63/UE and were approved by the committee for the Care and Use of Laboratory Animals of the Government of Upper Bavaria.

The mouse line *CRH*^*FKBP5−/−*^was generated at the Max Planck Institute of Psychiatry’s in-house breeding facility by crossing mice with a floxed Fkbp5 exon 9 (Fkbp5^lox/lox^) with mice expressing Cre under the CRH promoter (CRH^Cre^) [21, 30, 39]. Conditional *CRH*^*FKBP5−/−*^ animals lack FKBP51 in CRH-expressing neurons, while Cre-negative Fkbp5^lox/lox^ littermates (wt) served as controls. Experiments were performed on males aged 3–8 months, with equal representation of different ages in each group. Experimental conditions included exposure to chronic social defeat (CSDS) or daily handled control (ctrl). The resulting experimental groups were: wt ctrl, wt CSDS, *CRH*^*FKBP5−/−*^ ctrl, *CRH*^*FKBP5−/−*^ CSDS. RNAscope specificity verification was performed in *CRH-Venus* mice, a validated CRH knockout line [40].

All animals were habituated for one week after transportation to the local facilities and housed in individually ventilated cages (IVC; 30 cm x 16 cm x 16 cm; 501 cm^2^) serviced by a central airflow system (Tecniplast, IVC Green Line -GM500). Animals had *ad libidum* access to water and regular laboratory chow diet. They were maintained under constant environmental conditions with 12:12 hr light and dark cycle and with 23 ± 2 °C, and humidity of 55%). All IVC cages were supplied with sufficient bedding and nesting material. All cages had a plastic divider in order to prevent direct contact between the two housed animals.

### Experimental design

**Fig. 1:**
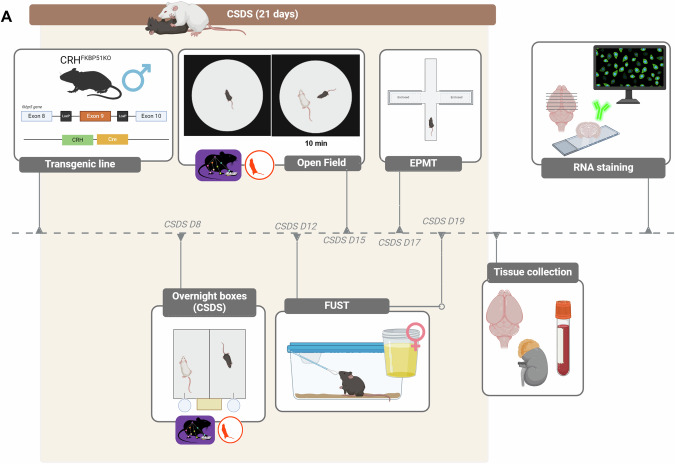
Schematic illustration of experimental timeline. **A** Behavioral testing commenced throughout the chronic stress procedure in the following order: over-night home-cage behavior (CSDS day 8), 1st Female Urine Sniffing Test (CSDS day 12), social interaction task (CSDS day 15), elevated plus maze, (CSDS day 17), 2^nd^ Female Urine Sniffing Test (CSDS day 19). Unstressed controls were tested at the corresponding time points. All animals were sacrificed one day after the last day CSDS in the morning. Brains, blood, and adrenals were extracted for subsequent analyses.

All experimental animals of each genotype were divided into two groups (ctrl or CSDS) in a semi-randomized fashion (wt ctrl=10, wt CSDS = 8, *CRH*^*FKBP5−/−*^ ctrl = 12, *CRH*^*FKBP5−/−*^ CSDS = 7). The data analysis and execution of experiments were performed blinded to the genotype of the animals. An overview of the experimental design is depicted in Fig. 1.

### Chronic Social Defeat paradigm (CSDS)

The CSDS paradigm consisted of exposing the experimental mice to an aggressive CD1 mouse for 21 consecutive days, as previously described [10, 41]. Detailed description can be found in the Supplementary Materials.

### Behavioral tests and analysis

All behavioral tests were recorded with top-view cameras using ANY-maze video tracking system software (RRID:SCR_014289, ver. 7.20, Stoelting Co.). The same software was also utilized for tracking overall locomotion in the open field arena. The FUST and EPM were automatically tracked and analyzed with ANY-maze. For open field and over-night tests, the videos were processed with open source pose-estimation software DeepLabCut (DLC, ver. 2.2.1) [15]. A 14-labels model marking the body parts and the open source behavioral analysis package DeepOF [10, 42] were utilized to calculate behavioral classifiers in both the open field arena and during selected time points in the over-night recordings.

### Female Urine Sniffing Test (FUST)

Urine samples were collected and pooled from 10 C57BL/6n females (age 3 months) immediately prior the experiment. The procedure followed protocol as previously described [43] (Supplementary Materials). To validate the stability of the effect elicited by CSDS, the FUST test was performed twice with one week between.

### Elevated Plus Maze (EPM)

A standardized version of the EPM with two closed and two open arms in opposing positions was implemented as described previously [44, 45] (Supplementary Materials).

### Open field and Social interaction task

The open field (OF) test involved initially allowing an animal to explore a round arena (⌀38 cm) with bedding for 10 min [10]. Following this, for the Social interaction (SI) phase, a non-aggressive novel 8-week old CD1 male was introduced into the arena for additional 10 min. Repeated use of the CD1s was avoided. Recordings were subsequently analyzed to capture both individual (wall-climbing, stopped-and-huddled, look-around, sniffing, and speed) and social behaviors (nose-to-nose, side-by-side, side-reverse-side, nose-to-tail, nose-to-body) [10].

### Overnight behavioral monitoring

To explore long-term changes in individual and social behaviors, mice were placed overnight (12 h) in customized cages (25 L x 25 W x 33H cm) with a perforated plexiglass divide, and *ad libitum* access to food and water. The animals in the CSDS condition were paired with their CD1 aggressor and the controls with their cage mate of the same genotype. Animals in both conditions remained separated with the plexiglass to avoid direct body to body contact and causalities. The top of the cage remained open to ensure undisturbed recording. Infrared lightbulbs were utilized for illumination to enhance recording clarity.

### Tissue sampling

On the final day between 8 and 12 am, the animals were weighed, deeply anesthetized with isoflurane, and sacrificed by decapitation. Trunk blood was collected in labelled 1.3 EDTA-coated microcentrifuge tubes and kept on ice until centrifugation (4 ^o^C, 8000 rpm for 15 mins). Plasma was then aliquoted into new labeled 0.5 ml tubes and stored at −80 ^o^C until corticosterone concentration (ng/mL) detection with an ELISA kit (Tecan, RE52211). Adrenals were dissected, stored in saline solution, cleared of adipose tissue and immediately weighted to assess weight differences relative to body weight. Brains were extracted, shock frozen in methyl butane on dry ice and stored at −80 ^o^C until sectioning. Coronal sections of 20 µm thickness were cut on a cryostat and stored at −20 ^o^C until staining. Regions of interest included prefrontal cortex (PFC), bed nucleus stria terminalis (BNST), paraventricular nucleus (PVN), central amygdala (ceAMY), dorsal hippocampus (dHIP).

### Multiplex RNA in-situ hybridization

The RNAscope^TM^ Multiplex Fluorescent Reagent Kit (ACD Biotecne Bio) was used to stain for FKBP51 (mm-Fkbp5-C1) and CRH (mm-CRH-C2) colocalized mRNA markers against a DAPI background. The protocol was followed according to the manufacturer.

### Z-score calculation

The z-scores are calculated as the mean of the behavioral observation of the wt control (${{\boldsymbol{\mu}}}$) subtracted from the observation of behavior (${{\boldsymbol{x}}}$), divided by the standard deviation of the control group (${{\boldsymbol{\sigma}}}$), following the formula: ${{\boldsymbol{Z}}}=\frac{{{\boldsymbol{X}}}{{\boldsymbol{-}}}{{\boldsymbol{\mu }}}}{{{\boldsymbol{\sigma }}}}$ [46]. For exploratory behavior characterization, an exploratory index was calculated incorporating “climbing” and “sniffing” with positive loading, and “huddling” and “lookaround” with negative loading, adjusted by multiplying with -1 to correct for behavior directionality. To characterize social behavior, a social interaction score was calculated including “nose-to-nose”, “side-by-side”, “side-re-side”, “nose-to-tail”, ”nose-to-body” with positive loading. The final z-score was derived by combining the different z-scores and accounting for the total number of tests: ${{{\boldsymbol{Z}}}}_{{{\boldsymbol{total}}}} = \frac{\,{\sum }_{{{\boldsymbol{1}}}}^{{{\boldsymbol{i}}}}{{\boldsymbol{z}}}{{{\boldsymbol{test}}}}_{{{\boldsymbol{i}}}}}{{{\boldsymbol{Number}}}\; {{\boldsymbol{of}}}\; {{\boldsymbol{tests}}}}$ [10].

The z-score of stress physiology has been also calculated with the relative adrenal weight and the body weight from the last day of the experimental timeline [10].

### Statistical analysis

Statistical analyses and graphs were conducted using GraphPad (v9.3.1., GraphPad Software, San Diego, California, USA) or Python (v 3.9.13). The data are presented as means ± standard error of the mean (SEM), with sample sizes indicated in the main text. Outliers were evaluated using a two-sided Grubb’s outlier test from the online GraphPad outlier calculator. All data were included for statistical analysis unless otherwise specified. Assumptions of normality and homoscedasticity were assessed using the Shapiro-Wilk test and QQ-plots and Lavene’s test, respectively. Data violating these assumptions were analyzed using non-parametric tests. The body weight data were analyzed using 3-way repeated measures ANOVA with genotype, condition as independent variables and body weight as dependent variable. The comparisons across conditions per behavioral or molecular parameter were compared by using 2-way ANOVA, followed by Bonferroni post hoc tests. Statistical significance was considered as *p* < 0.05 for all main and post hoc effects, and *p* < 0.1 for ANOVA interaction effects. Further significance was defined as *p* < 0.01(**), *p* < 0.001(***), and *p* < 0.0001(****).

## Results

### Line validation

**Fig. 2:**
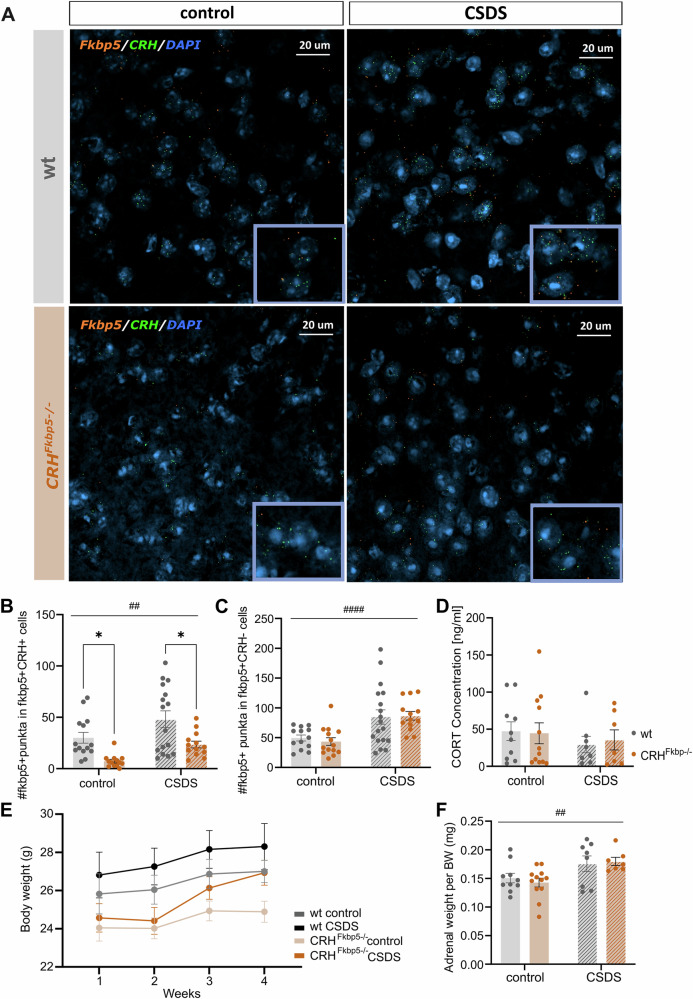
Verification of the KO line and characterization of body weight, adrenal weight and CORT levels. **A** The representative images are taken from the paraventricular nucleus (PVN) region of the mouse brain with a magnification of 20x confocal microscope objective. **B** The quantification points in the graphs represent *Fkbp5* expression in *Crh* positive cells from pooled regions with CRH expression: dHipp, ceAmy, BNST, PVN, and PFC. A significant reduction of raw count of double positive cells (*Crh* and *Fkbp5*) was observed between the KO-line (*p* < 0.001) and wt (*p* < 0.001). This reduction was notable in both control and CSDS exposed animals. (stress effect: #=*p* < *0.05*, ##=*p* < *0.01*, ###=*p* < *0.001, ####=p* < *0.0001*, genotype effect: *=p < *0.05).* **C** The specificity of the KO has been validated by counting the positive puncta of *Fkbp5* in *Crh* negative cells from the aforementioned regions. With significantly elevated *Fkbp5* levels in the CSDS condition (*p* < *0.0001*), but no differences between the two genotype groups. **D** Baseline corticosterone (CORT) levels marked no differences between stress conditions nor genotype. **E** The body weight of the animals marked an overall increase over the experimental timeline. There was a main effect of genotype, without post-hoc tests reaching significant differences. **F** The adrenal weight of CSDS animals was significantly elevated (*p* < *0.01*) but no effect of genotype was marked.

The *CRH*^*FKBP5−/−*^ mouse line was generated to study the effects of FKBP51 in CRH-expressing neurons on the behavior and stress resilience. We quantified *Fkbp5*^*+*^ and *Crh*^+^ neuron co-expression in CRH-rich regions: PFC, BNST, PVN, ceAMY, dHIP (Fig. 2A). The CRH RNAscope specificity was validated in CRH knockout mice from the *CRH-Venus* line (Supplementary Fig. 1A). This quantification revealed significantly fewer of *Fkbp5* mRNA puncta in *Crh*^*+*^ neurons across all regions in the *CRH*^*FKBP5−/−*^ animals under both conditions (genotype: *F*_*(1,54)*_ = 17.45, *p* = 0.0001, CSDS *F*_*(1,54)*_ = 9.149, *p* = 0.0039, interaction *F*_*(1,54)*_ = 0.02586, *p* = 0.8729) (Fig. 2B). *Fkbp5* mRNA puncta levels in *Crh*^*−*^ cells did not differ between genotypes, with marked elevation only associated with CSDS exposure. Confirming high FKBP5 deletion specificity (genotype: *F*_*(1.54)*_ = 0.04694, *p* = 0.8293, CSDS: *F*_*(1,54)*_ = 18.48, *p* < 0.0001, interaction *F*_*(1,54)*_ = 0.1979, *p* = 0.6582) (Fig. 2C). The overall *Crh* puncta in *fkbp5- cells* were also affected only by stress exposure but not the genotype (genotype: *F*_*(1,54)*_ = 0.001327, *p* = 0.9711, CSDS *F*_*(1,54)*_ = 8.748, *p* = 0.0048, interaction *F*_*(1,54)*_ = 1.774, *p* = 0.1892) (Supplementary Fig. 1B).

### Physiological parameters are unaffected by loss of FKBP51 in CRH-neurons

We assessed physiological parameters under basal and CSDS conditions. Corticosterone (CORT) levels did not differ significantly between groups (genotype *F*_*(1,33)*_ = 0.01556, *p* = 0.9015, CSDS *F*_*(1,33)*_ = 0.9616, *p* = 0.3339, interaction *F*_*(1,33)*_ = 0.1057, *p* = 0.7472) (Fig. 2D). Similarly, starting body weight and body weight gain throughout the experiment were not significantly altered by the condition. There was a main effect of genotype, without post-hoc tests reaching significant differences (Time *F*_*1.271,22.87*_ = 16.42, *p* = 0.0002, CSDS: *F*_*1,42*_ = 1.870, *p* = 0.1787, genotype: *F*_*1,18*_ = 9.461, *p* = 0.0065, Time x CSDS *F*_*3,42*_ = 1.272, *p* = 0.2963, Time x genotype *F*_*2.651,37.12*_ = 0.9093, *p* = 0.4357; CSDS x Genotype *F*_*1,42*_ = 0.07341, *p* = 0.7878, Time x CSDS x genotype *F*_*3,42*_ = 1.390, *p* = 0.2590) (Fig. 2E). Adrenal weights marked a significant increase in the CSDS animals (CSDS *F*_*(1,33)*_ = 10.50, *p* = 0.0027), independent of genotype (genotype *F*_*(1,33)*_ = 0.03726, *p* = 0.8481, interaction *F*_*(1,33)*_ = 0.4003, *p* = 0.5314) (Fig. 2F).

### Behavioral differences driven by loss of FKBP51 in CRH neurons are dependent on social context

**Fig. 3:**
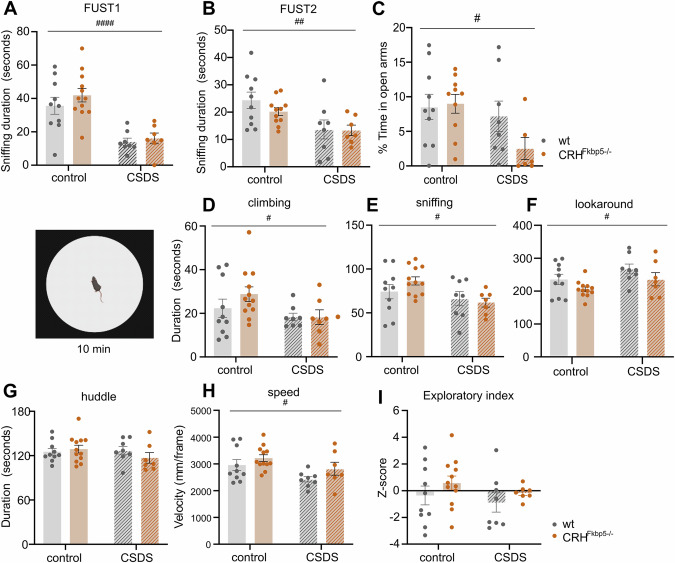
The CSDS animals demonstrated chronically increased anhedonia and consistent anxious-like set of behaviors during elevated plus maze (EPM) and exploration of the open field arena. **A**, **B** The female urine sniffing test (FUST) revealed significant association of shorter sniffing time in the CSDS group during both instances of testing (*p* < *0.001*) (stress effect: #=*p* < *0.05*, ##=*p* < *0.01*, ###=*p* < *0.001, ####=p* < *0.0001)*. **C** Overall animals subjected to the CSDS demonstrated significantly decreased preference for the open arms (*p* < *0.01*) of the EPM. **D**−**G** In the open field paradigm, general effect of stress (*p* < 0.05) was observed on almost all of the behavioral parameters except huddle. The significantly increased duration of looking around (*p* < 0.05) in the CSDS animals, can be interpreted as an indicator of anxious-like pattern and suggest a reduced exploratory behavior of these animals. No significant differences were observed on the level of genotype. **H** The speed parameter was significantly decreased in the CSDS group, regardless of genotype (*p* < 0.05). **I** The overall exploratory index of the open field arena did not show any differences between the groups.

We assessed the classical behavioral hallmarks of CSDS, including anhedonia and anxiety. The FUST detected consistently decreased sniffing times of the CSDS animals (CSDS: “FUST1” *F*_*(1,33)*_ = 31.78, *p* < 0.0001 ; “FUST2”: *F*_*(1,33)*_ = 11.61, *p* = 0.0017;), with no genotype differences (genotype: “FUST1”: *F*_*(1,33)*_ = 0.9706, *p* = 0.3317, interaction *F*_*(1,33)*_ = 0.2539, *p* = 0.6177; “FUST2”: *F*_*(1,33)*_ = 0.1766, *p* = 0.6771, interaction *F*_*(1,33)*_ = 0.09695, *p* = 0.7575) (Fig. 3A, B). For anxiety-related behavior, the CSDS animals spent significantly reduced time percentage in the EPM’s open arms (CSDS: *F*_*(1,30)*_ = 4.617, *p* = 0.0398), without genotype differences (genotype: *F*_*(1,30)*_ = 1.344, *p* = 0.2555, interaction: *F*_*(1,30)*_ = 1.978, *p* = 0.1699) (Fig. 3C). The total distance travelled in the EPM was significantly lower for the stressed group without an effect of genotype (CSDS: *F*_*(1.33)*_ = 8.922, *p* = 0.0056, genotype: *F*_*(1.33)*_ = 1.110, *p* = 0.3005, interaction: *F*_*(1.33)*_ = 0.1062, *p* = 0.7468) (Supplementary Fig. 1C).

Next, we assessed individual and social behavioral alterations in the OF and SI. The OF analysis showed consistent alterations in individual behavior in CSDS animals (CSDS: “climbing”: *F*_*(1,33)*_ = 4.205, *p* = 0.0483; “sniffing”: *F*_*(1,33)*_ = 5.735, *p* = 0.0225; “lookaround”: *F*_*(1,33)*_ = 4.897, *p* = 0.0339; “huddle”: *F*_*(1,33)*_ = 0.8258, *p* = 0.3701; “speed”: *F*_*(1,33)*_ = 7.190, *p* = 0.0114), with in most cases no effect of genotype or interaction (genotype: “climbing”: *F*_*(1.33)*_ = 0.7968, *p* = 0.3785; “sniffing”: *F*_*(1,33)*_ = 0.3186, *p* = 0.5763; “huddle”: *F*_*(1,33)*_ = 0.3052, *p* = 0.5843; “speed”: *F*_*(1,33)*_ = 3.527, *p* = 0.0692; interaction: “climbing”: *F*_*(1,33)*_ = 0.8510, *p* = 0.3630; “sniffing”: *F*_*(1,33)*_ = 1.480, *p* = 0.2323; “lookaround”: *F*_*(1,33)*_ = 0.003421, *p* = 0.9537; “huddle”: *F*_*(1,33)*_ = 1.419, *p* = 0.2420; “speed”: *F*_*(1,33)*_ = 0.1580, *p* = 0.6936) (Fig. 3D–H). For the parameter “lookaround” we detected a significant effect of genotype (“lookaround”: *F*_*(1,33)*_ = 4.665, *p* = 0.0381). The exploratory index calculation did not reveal any significant differences (CSDS: *F*_*(1,33)*_ = 1.018, *p* = 0.3203, genotype *F*_*(1,33)*_ = 1.788, *p* = 0.1903, interaction: *F*_*(1,33)*_ = 0.02771, *p* = 0.8688) (Fig. 3I). The conventional measure total distance travelled in the open field confirmed the general stress phenotype but did not reveal any genotype differences between the animals (CSDS: *F*_*(1.33)*_ = 5.442, *p* = 0.0259, genotype: *F*_*(1.33)*_ = 3.764, *p* = 0.0610, interaction: *F*_*(1.33)*_ = 0.2028, *p* = 0.6554) (Supplementary Fig. 1D). Similar findings were revealed also for the time spent in the center of arena (genotype: *F*_*(1,33)*_ = 0.004254, *p* = 0.9484, CSDS: *F*_*(1,33)*_ = 5.906, *p* = 0.0207, interaction: *F*_*(1,33)*_ = 0.007405, *p* = 0.9319) (Supplementary Fig. 1E).

**Fig. 4:**
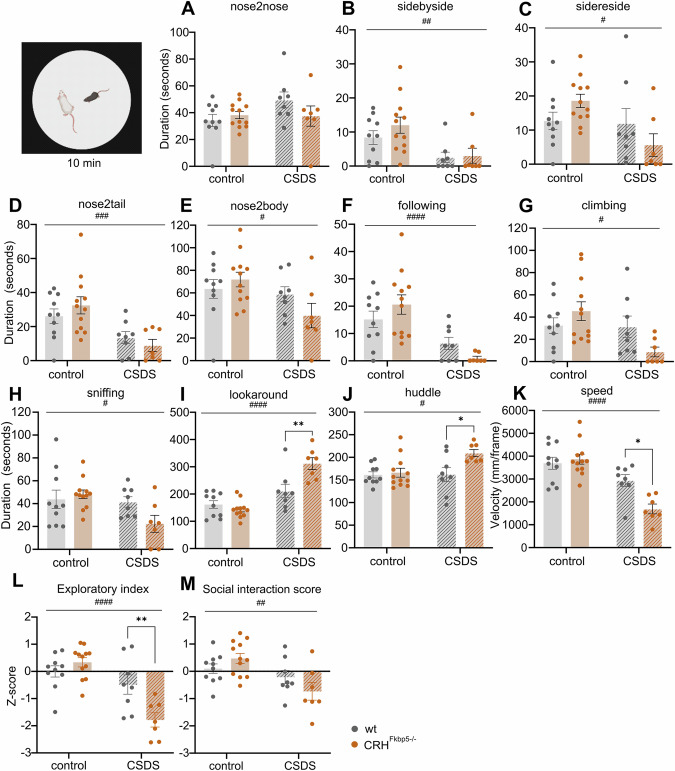
Significant differences in individual and social behaviors between transgenic animals in the CSDS condition arise, when novel CD1 is introduced in the social interaction task. **A**–**F** The majority of the social behavioral parameters were overall significantly decreased in the CSDS animals (stress effect: #=*p* < *0.05*, ##=*p* < *0.01*, ###=*p* < *0.001, ####=p* < *0.0001*, genotype effect: *=p < 0.05, **=p < *0.01)*. **G**–**J** The individual parameters marked a significant decrease in the CSDS group. With emerging genotype differences in looking around and huddling behaviors (*p* < *0.05)* **K** Overall impact of stress was again seen for speed parameter (*p* < *0*.05), with the CRH^*fkbp5−/−*^ animals moving the least of all the depicted groups (*p* < *0.05*). **L** An exploratory index confirmed the overall effect of stress and within the CSDS group also indicated a significant genotype difference, with the CSDS CRH^*fkbp5−/−*^ animals demonstrating significantly the least set of active exploratory behaviors in the arena with a non-aggressive CD1 animal. **M** The social interaction score solidified the effect of stress, by depicting the overall decreased social contact of the CSDS animals with the novel CD1 animal (*p* < *0.05*).

Interestingly, the novel CD1 in the SI yielded more pronounced differences in both social (Fig. 4A−F) and individual behaviors (Fig. 4G–K), except for “nosetonose” interaction (CSDS: *F*_*(1,33)*_ = 2.175, *p* = 0.1497; genotype: *F*_*(1,33)*_ = 0.6677, *p* = 0.4197; interaction: *F*_*(1,33)*_ = 2.603, *p* = 0.1162) (Fig. 4A). The social behaviors significantly decreased in CSDS animals (CSDS: “sidebyside” *F*_*(1,33)*_ = 11.09, *p* = 0.0021, “sidereside” *F*_*(1,33)*_ = 5.217, *p* = 0.0289, “nose2tail” CSDS: *F*_*(1,33)*_ = 15.46, *p* = 0.0004, “nose2body” *F*_*(1,33)*_ = 5.120, *p* = 0.0304, “following” *F*_*(1,33)*_ = 21.32, *p* < 0.0001), independent of FKBP51 deletion (genotype: “sidebyside” *F*_*(1,33)*_ = 0.8558, *p* = 0.3616, “sidereside” *F*_*(1,33)*_ = 0.002906, *p* = 0.9573, “nose2tail” *F*_*(1,33)*_ = 1.424, *p* = 0.2412, “nose2body” *F*_*(1,33)*_ = 0.4372, *p* = 0.5130, “following” *F*_*(1,33)*_ = 0.0003417, *p* = 0.9852, interaction: “sidebyside” *F*_*(1,33)*_ = 0.5182, *p* = 0.4767, “sidereside” *F*_*(1,33)*_ = 4.024, *p* = 0.0531, “nose2tail” *F*_*(1,33)*_ = 0.4758, *p* = 0.4953, “nose2body” *F*_*(1,33)*_ = 2.873, *p* = 0.0995, “following” *F*_*(1,33)*_ = 3.063, *p* = 0.0894) (Fig. 4B–F).

Intriguingly, the individual behaviors in a social context were affected not only by stress but also by genotype. Highlighting lookaround, huddle and speed, where the stress-induced effect was significantly elevated in *CRH*^*FKBP5KO*^ mice versus the wt (CSDS: “lookaround” *F*_*(1,33)*_ = 41.54, *p* < 0.0001, “huddle” *F*_*(1,33)*_ = 4.244, *p* = 0.0473, “speed” *F*_*(1,33)*_ = 34.84, *p* < 0.0001; genotype: “lookaround” *F*_*(1,33)*_ = 5.906, *p* = 0.0207, “huddle” *F*_*(1,33)*_ = 5.915, *p* = 0.0206, “speed” *F*_*(1,33)*_ = 4.621, *p* = 0.0390; interaction: “lookaround” *F*_*(1,33)*_ = 12.35, *p* = 0.0013, “huddle” *F*_*(1,33)*_ = 3.932, *p* = 0.0558, “speed” *F*_*(1,33)*_ = 8.199, *p* = 0.0072) (Fig. 4I–K). The stress effect persisted in the other behaviours, but without genotype effect (CSDS: “sniffing” *F*_*(1,33)*_ = 5.182, *p* = 0.0294, “climbing” *F*_*(1,33)*_ = 5.266, *p* = 0.00282, genotype: “sniffing” *F*_*(1,33)*_ = 1.421, *p* = 0.2418, “climbing” *F*_*(1,33)*_ = 0.3452, *p* = 0.5609, interaction: “sniffing” *F*_*(1,33)*_ = 3.617, *p* = 0.0659, “climbing” *F*_*(1,33)*_ = 4.710, *p* = 0.0373) (Fig. 4G, H). The *CRH*^*FKBP5−/−*^ animals showed the most significant reduction in the exploratory index compared to the wt in the CSDS condition (CSDS: *F*_*(1,33)*_ = 27.93, *p* < 0.0001, genotype: *F*_*(1,33)*_ = 3.844, *p* = 0.0584; interaction: *F*_*(1,33)*_ = 11.05, *p* = 0.0022) (Fig. 4L). The social interaction score was significantly lower for the CSDS animals with an interaction influence (CSDS: *F*_*(1,33)*_ = 11.26, *p* = 0.0020; genotype: *F*_*(1,33)*_ = 0.1047, *p* = 0.7483; interaction: *F*_*(1,33)*_ = 3.896, *p* = 0.0568) (Fig. 4M).

A PCA analysis of the behaviors in the SI task identified two principal components (PC) with 28.9% and 20% variance explained, respectively (Supplementary Fig. 1F). The PCA showed a significant effect of the CSDS condition with clear separation of the stressed and control cohorts (genotype: *F*_*(1,33)*_ = 0.104, *p* = 0.749, CSDS: *F*_*(1,33)*_ = 27.754, *p* < 0.001, interaction: *F*_*(1,33)*_ = 0.127, *p* = 0.724) (Supplementary Fig. 1G), with PC1 eigenvalues contributing significantly to this stress distinction (CSDS: *F*_*(1,33)*_ = 29.69, *p* < 0.001) (Supplementary Fig. 1H). The top five contributing behaviors essential for identifying the stress phenotype consisted of B-speed, B-lookaround, B-nose-to-tail, W-nose-to-tail, and W-following (Supplementary Fig. 1I). The eigenvalue for PC2 did not reach significance in explaining the CSDS distinction (CSDS: *F*_*(1,33)*_ = 0.026, *p* = 0.874) and the top five behaviors included W-nose-to-body, W-following, B-sniffing, side-reverse-side, and W-sniffing (Supplementary Fig. 1J-K). This composed of mainly behaviors from the CD1 animals and loaded with lower scores. The “B” indicates the behaviors of our black experimental animals and “W” refers to the behaviors of the white CD1s.

The z-score of stress physiology is regarded as a strong CSDS profiling tool and was used for correlation analysis between the SI task’s top-ranking behaviors identified in the PCA analysis [10]. As demonstrated by Bordes et al. 2023, we also observed strongly significant positive correlation (*R* = 0.4986, *p* = 0.0020) (Supplementary Fig. 1L) [10].

### Chronic stress affects individual and social behavior in the home cage

**Fig. 5:**
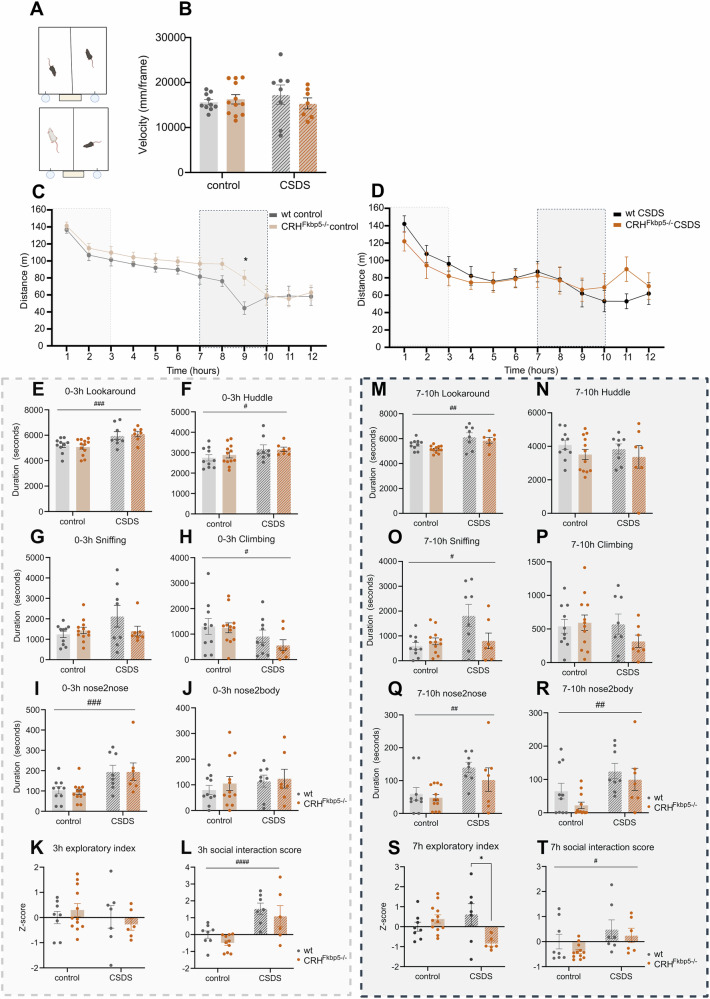
Overnight behavioral classifiers analysis provides an insight into longitudinal interaction between the CD1 and experimental animals in a home-cage-like environment. **A** Illustration of the experimental set-up of the overnight-recording paradigm, for either CD1 and experimental animal or two littermates. In both cases, divided in the middle with perforated plexiglass. **B** No significant changes in velocity were detected over the 12 hours between the groups. **C** Comparison of distance travelled between the genotypes of control group. Significant difference was observed during 9th hour of recording (*p* < 0.05) (stress effect: #=*p* < *0.05*, ##=*p* < *0.01*, ###=*p* < *0.001, ####=p* < *0.0001*, genotype effect: *=p < *0.05)*. **D** Comparison of distance travelled between the genotypes within the CSDS group, the deviating patterns did not reach significance level. **E**–**J** Behavioral classifiers of the first 0–3 h of recording. We can observe significant upregulation of looking around behavior (*p* < *0.01*) together with nose to nose interaction (*p* < *0.01*) and downregulation of climbing (*p* < *0.05*) in the CSDS group in comparison to the controls. **K** The exploratory index over this period showed to be similar across all the groups. **L** The social score illustrates an increase in social activity of the CSDS group, irrespective of genotype. (**M**-**R**) The analysis of behavior from the time window 7–10 h showed preservation of upregulated looking around behavior (*p* < *0.01*) and nose to nose interaction (*p* < *0.01*). Additional parameters were marked as significant during this time, being sniffing behavior (*p* < *0.05*) and nose to body (*p* < *0.05*). **S** The exploratory index from 7 to 10 h period showed this time a significant effect only in the CSDS group, where CRH^*fkbp5−/−*^ animals showed a significant decreased exploration in comparison to their wt counterparts. **T** The social score remained to be significantly upregulated also at this later stage in the observation period (*p* < *0.05)*.

Lastly, we also investigated the effects of stress exposure and loss of FKBP51 in CRH neurons on home cage behavior during the activity phase (Fig. 5A). Throughout the night, the cumulative speed did not differ between the groups (CSDS: *F*_*(1,33)*_ = 0.08689, *p* = 0.7700, genotype: *F*_*(1,33)*_ = 0.2525, *p* = 6186, interaction: *F*_*(1,33)*_ = 0.9988, *p* = 0.3249, Fig. 5B). Analyzing the temporal dynamics of distance traveled in hourly bouts revealed an overall decrease across the recording period for all groups (3-way ANOVA: within subject effect of time: *F*_*(11,220)*_ = 27.32, *p* < 0.0001) (Fig. 5C, D). This decrease was significantly more prominent in *CRH*^*FKBP5−/−*^ mice under non-stressed conditions compared to their wild-type littermates in the second half of the night, especially at the time mark of 9 hours (genotype: *F*_*(11,220)*_ = 38.58, *p* < 0.0001 & post-hoc: hour 9 (*p* = 0.0168), Fig. 5C). Under CSDS conditions, this effect was not detectable (CSDS: *F*_*(11,143)*_ = 7.729, *p* < 0.0001 & post-hoc: hour9 (p > 0.9999, Fig. 5D).

To further assess individual and social behavior, we selected two temporal windows, based on circadian locomotion pattern, for further analysis: the 0–3 h window at the beginning of the activity (Fig. 5E–L), and the 7–10 h window (Fig. 5M–R), where differences in speed were apparent. In the 0–3 h window, DeepOF analysis revealed again significant effects of CSDS on individual (CSDS: “lookaround” *F*_*(1,33)*_ = 16.25, *p* = 0.0003, “huddle” *F*_*(1,33)*_ = 4.523, *p* = 0.0410, “climbing” *F*_*(1,33)*_ = 4.476, *p* = 0.0418, Fig. 5E, F, H) as well as social behavior (CSDS: “nose2nose” *F*_*(1,33)*_ = 14.03, *p* = 0.0007, Fig. 5I). Here, no main effects of genotype were apparent (genotype: “lookaround” *F*_*(1,33)*_ = 0.00009, *p* = 0.9761, “huddle” *F*_*(1,33)*_ = 0.1454, *p* = 0.1454, “sniffing” *F*_*(1,33)*_ = 0.8966, *p* = 0.3506, “climbing” *F*_*(1,33)*_ = 0.6312, *p* = 0.4324, “nose2nose” *F*_*(1,33)*_ = 0.02541, *p* = 0.8743, “nose2body” *F*_*(1,33)*_ = 0.4135, *p* = 0.5246, interaction: “lookaround” *F*_*(1,33)*_ = 0.3180, *p* = 0.5766, “huddle” *F*_*(1,33)*_ = 0.2158, *p* = 0.6453, “sniffing” *F*_*(1,33)*_ = 2.634, *p* = 0.1141, “climbing” *F*_*(1,33)*_ = 0.3351, *p* = 0.5665, “nose2nose” *F*_*(1,33)*_ = 0.02588, *p* = 0.8732, “nose2body” *F*_*(1,33)*_ = 0.08390, *p* = 0.7739) (Fig. 5E–J). This was further confirmed by z-scores, where specifically the social interaction score substantially increased with the CSDS effect, independently of genotype (“3 h exploratory index” CSDS: *F*_*(1,33)*_ = 0.7777, *p* = 0.3849; genotype: *F*_*(1,33)*_ = 0.0011, *p* = 0.9737; interaction: *F*_*(1,33)*_ = 0.09411, *p* = 0.3398; “3 h social interaction score”: CSDS: *F*_*(1,33)*_ = 23.57, *p* = 0.0001; genotype: *F*_*(1,33)*_ = 3.081, *p* = 0.0898; interaction: *F*_*(1,33)*_ = 0.007407, *p* = 0.9320) (Fig. 5K, L).

During the 7–10 h time window, our analysis revealed significant stress and genotype effects (Fig. 5M–R). Notably, different patterns of individual and social interactions were significantly altered by CSDS exposure (CSDS: “lookaround” *F*_*(1,33)*_ = 12.33, *p* = 0.0013, “sniffing” *F*_*(1,33)*_ = 5.874, *p* = 0.0210, “nose2nose” *F*_*(1,33)*_ = 11.77, *p* = 0.0016, “nose2body” *F*_*(1,33)*_ = 9.932, *p* = 0.0034) (Fig. 5M, O, Q, R), regardless of the genotype (genotype: “lookaround” *F*_*(1,33)*_ = 2.946, *p* = 0.0955, “nose2nose” *F*_*(1,33)*_ = 1.533, *p* = 0.2244, “nose2body” *F*_*(1,33)*_ = 2.407, *p* = 0.1303, interaction: “lookaround” *F*_*(1,33)*_ = 0.06681, *p* = 0.7976, “nose2nose” *F*_*(1,33)*_ = 0.4118, *p* = 0.5255, “nose2body” *F*_*(1,33)*_ = 0.1672, *p* = 0.6852) and in the case of sniffing, the stress effect depended on the genotype (genotype: “sniffing” *F*_*(1,33)*_ = 2.617, *p* = 0.1153, interaction: “sniffing” *F*_*(1,33)*_ = 5.473, *p* = 0.0255). For the remaining behaviors no interactions or effects were detected (CSDS: “huddle” *F*_*(1,33)*_ = 0.2508, *p* = 0.6198, “climbing” *F*_*(1,33)*_ = 0.9964, *p* = 0.3254, genotype: “huddle” *F*_*(1,33)*_ = 1.858, *p* = 0.1820, “climbing” *F*_*(1,33)*_ = 0.6961, *p* = 0.4101, interaction: “huddle” *F*_*(1,33)*_ = 0.02039, *p* = 0.8873, “climbing” *F*_*(1,33)*_ = 1.578, *p* = 0.2179) (Fig. 5 N, P). Additionally, a significant interaction effect in the exploratory index was observed, where specifically under CSDS, *CRH*^*FKBP5−/−*^ mice displayed a significantly lower exploratory behavior compared to wt (CSDS: *F*_*(1,33)*_ = 0.7800, *p* = 0.3842, genotype: *F*_*(1,33)*_ = 2.705, *p* = 0.1105, interaction: *F*_*(1,33)*_ = 8.717, *p* = 0.0061) (Fig. 5S). For social interaction score, we again observed stark differences of CSDS animals (CSDS: *F*_*(1,33)*_ = 6.870, *p* = 0.0136), but no genotype or interaction effects (genotype: *F*_*(1,33)*_ = 0.8475, *p* = 0.3646, interaction: *F*_*(1,33)*_ = 0.4296, *p* = 0.5172) (Fig. 5T).

The interactions of the aggressive CD1 animals with their cage mate in the overnight recordings was unaffected by the genotype of the experimental animal across all parameters measured (Supplementary Fig. 2A–R).

## Discussion

This study delves into how the psychiatric risk factor FKBP51 influences neurons expressing the stress-related neuropeptide CRH, both under basal conditions and following a chronic stress exposure. Employing advanced deep phenotyping methods, we demonstrated that the lack of FKBP51 in this neuronal population leads to distinct alterations in naturalistic individual and social behaviors, implicating a heightened response to social stress environment.

Chronic stress leads to a number of physiological alterations such as body weight adjustments or increased adrenal weights [41], which were also observed in the current study, independent of genotype. FKBP51 deletion in all cells of the body was reported to lead to reduced body weight [47], and we here report that its specific deletion in CRH-expressing neurons produced a similar overall effect. This is in line with a previous study, where FKBP51 deletion in the PVN also reduced body weight in adult males [21]. On the level of corticosterone secretion, no differences were detected between the wild type and *CRH*^*FKBP5−/−*^ mice, which could be due to sampling exclusively under basal conditions. In addition, we have previously shown that overexpressing FKBP51 in *Sim1*-positive neurons of the PVN could only partially recapitulate the endocrine effects of a FKBP51 overexpression in all PVN neurons [21]. Furthermore, FKBP51 has different impact on HPA axis function in different brain regions, which is supported by our previous finding that FKBP51 deletion in the BNST in fact increases corticosterone secretion [31]. Thus, it is plausible that for specifically endocrine regulation deleting FKBP51 in all CRH-positive neurons of the brain results in effects that cancel each other out.

In addition, chronic stress also triggers a myriad behavioral adjustments in animals to cope with environmental challenges [8, 48]. Prior research on this topic primarily relied on simplistic tests, offering limited insight into how animals adapt to stress [49]. By combining detailed behavioral observations with chronic social stress exposure, we expanded on the known stress-induced behavioral changes, unveiling nuanced alterations. While traditional assessments still captured classic stress markers like anhedonia and the avoidance of the round open field’s center, observing social interactions in freely moving animals and monitoring behavior throughout the activity phase revealed nuanced, previously hidden alterations. Previous studies show that global FKBP5 knockout does not affect anxiety behavior neither in basal or stressed conditions [50–52]. However, amygdala specific knock-out reduced stress-induced anxiety, while BNST-specific knockout increased the occurrence of anxiety-like behaviors [31, 53]. We were able to show a dynamic shift in the profile of exploratory index behaviors in *CRH*^*FKBP5−/−*^ mice. This behavioral profile was in the SI task heavily anxiety-like and internalizing in nature, especially in the CSDS animals. A similar profile but without the genotype effect was also present in the presence of aggressor behind the divider in the overnight analysis. In the latter analyzed segment of the night, this exploratory profile shifted and was marked by reduced active exploration, such as sniffing, in the transgenic stressed animals. These subtle shifts, particularly evident in social contexts, underscore the importance of studying group dynamics alongside individual assessments. In our context, the lack of FKBP51 in CRH neurons triggers a specific set of behaviors, characterized by exaggerated anxiety-like response to conspecifics and failure to engage with the environment in the presence of chronic social stressor, potentially failing to perform avoidance or escape behaviors. This highlights how this genetic modification under stress conditions affects specific behavioral subtypes, providing insight into broader and crude underlying mechanism. Additionally, our study revealed that stress-induced behavioral changes unfolded dynamically over the activity phase, emphasizing the need for extended testing periods.

It is also important to note that assessments of freely moving animals pose methodological challenges, impacting the observed results. While we showed that the genotype-dependent differences in circadian social behavior are independent of behavior of the social CD1 counterpart, the strain and/or genotype of social partner or prior experiences can undoubtedly affect the exhibited social behavior. For our control group, the same genotype housed provided us with clearer interpretation, but undoubtedly poses a limitation in the complexity of social interactions that are in our case limited to same-genotype individuals in social groups. In the SI task, PCA analysis identified a principal component, which was predominantly characterized by the behaviors of the non-aggressive CD1 animal. However, the eigenvalues of this principal component did not significantly explain the variance between the conditions. Further consideration focuses on the specific experimental settings, such as cage dividers in the social defeat paradigm as they reduce the complexity of potential social interactions. Lastly, all behavioral observations in traditional behavioral tasks are it is notoriously difficult and nearly impossible to allow for distinguishing between an animal’s motivation to perform a specific behavior and its overall response to environmental stimuli, in our context social stressors. The social engagement and avoidance fluctuations could stem either directly from being differentially impacted by the stress exposure or from unrelated secondary mechanisms to the initial stress response. Altogether, our current results highlight the knowledge gain we can leverage from implementing novel approaches to behavioral phenotyping and acknowledge the remaining challenges in behavioral phenotyping.

Both the neuropeptide CRH and the GR co-chaperone FKBP51 have been implicated in mediating alterations in chronic stress [51, 54]. However, their function is profoundly influenced by the cell type and brain region, with the same alteration promoting resilience in one context and vulnerability in another [21, 29, 31, 34, 35]. Our study on FKBP51 in CRH-expressing neurons highlight this notion and reveals a heightened response to chronic social stress in mice lacking FKBP51 in CRH-positive neurons. This observation, particularly evident in unrestricted social contexts, where the presence of another conspecific further enhanced alterations in non-social exploratory behavior, including reduced speed and enhanced vigilance behavior as huddling and lookaround. These observations suggest heightened anxiety and stress reactivity in context, where social presence acts as a potent additional stressor. Importantly, these behavioral alterations of CRH-specific FKBP51 knockout mice are not readily evident in classical physiological or behavioral readouts of chronic stress exposure.

The behavioral profile of the CRH-specific FKBP51 knock-out mice also dynamically unveils with time. In the overnight tracking, the analyzed segment between 7 and 10 h showed significant decrease in exploratory behaviors of the stressed animals lacking FKBP51 in the CRH expressing cells, pointing towards the circadian rhythms in gene expression and neuronal activity of CRH neurons. This was previously detailed in the context of PVN^CRH^ and description of their peak clock gene rhythms peaking at midday and reaching their nadir in early morning hours [55]. Together with suprachiasmatic nucleus neurons releasing vasoactive intestinal peptide, they govern glucocorticoid release and generate the daily surge in corticosterone before waking [55]. Our findings suggest that in this concert of actions governing the glucocorticoid release, FKBP51 might play a moderating role when animal is exposed to a chronic stressor. Therefore, if ablated, the decrease in CRH and increase in CORT in this manner without co-expressed FKBP51, might results in more pronounced effects of present stressors on exploratory behaviors.

Mechanistically, FKBP51 has a multitude of possibilities to modulate the function of CRH-expressing neurons. Exemplarily, FKBP51 acts as a co-chaperone for GRs, and its absence results in a higher GR sensitivity and consequently altered GR-dependent gene expression profiles in CRH-expressing neurons. Conversely, FKBP51 overexpression can also modify neuronal excitability and function, as previously reported in the PVN CRH neurons [21]. Moreover, FKBP51 has been described to play a decisive role in GR-independent protein-protein interactions. Hartmann and colleagues recently demonstrated that secretory autophagy is regulated by FKBP51 in interaction with spindle and kinetochore-associated protein 2 (SKA2), thereby affecting neuroinflammation and behavior [56]. Importantly, as both FKBP51 and CRH have been implicated in mediating the response to antidepressants, it is possible that there are also converging signaling pathways mediating these effects [57, 58].

This study prompts further directions for follow-up research. While we have emphasized the importance of investigating FKBP51 KO on social behavior in CRH^+^ cells, it is crucial to acknowledge sex differences in the stress response. Our focus on male mice limits extrapolation to females, necessitating further sex-dependent studies [59, 60]. Additionally, CRH expression in diverse neuronal cell types and brain regions may contribute to distinct behavioral phenotypes, which our genetic approach may not fully capture [35, 36]. Additionally, the utilization of unsupervised behavioral analyses pipelines would be beneficial in detecting obscured behavioral phenotypes. The adaptation of these still poses a challenge in complex gene by environment interactions and represents an interesting prospect for further development [10]. Finally, we here focused on a thorough behavioral assessment of the consequences of chronic stress exposure, omitting more detailed insights in neuronal structure, function or molecular alterations.

Taken together, the current research demonstrates the criticality of cell-type specificity and context dependence in understanding stress behavioral responses. It urges a shift from broad-brushstroke approaches to meticulously dissecting the interplay between molecules, cell types, and social interactions. The inclusion of deep phenotyping technologies holds immense potential for unlocking individual vulnerabilities and paving the way for highly targeted interventions to promote stress resilience. In essence, this study unveils the intricate “lock and key” nature of stress responses, where the impact of a single molecule like FKBP51 hinges on the specific cellular context. By deciphering these nuances, we can move beyond generic strategies and design personalized approaches to managing stress, ultimately fostering individual well-being.

## Supplementary information

Supplemental Material
