# Supplementary material for: Deep phenotyping reveals CRH and FKBP51-dependent behavioral profiles following chronic social stress exposure in male mice
Source: Neuropsychopharmacology. 2024 Oct 22;50(3):556–67. doi: 10.1038/s41386-024-02008-9 (PMC11736030; doi:10.1038/s41386-024-02008-9)
Supplement: Supplementary file 1 — Supplemental Material [file 41386_2024_2008_MOESM1_ESM.docx]

Deep phenotyping reveals CRH and FKBP51-dependent behavioral profiles following chronic social stress exposure in male mice

Veronika Kovarova, Joeri Bordes, Shiladitya Mitra, Sowmya Narayan, Margherita Springer, Lea Maria Brix, Jan M. Deussing, Mathias V. Schmidt

**Supplemental Material**

1. **Supplemental Methods**

**Chronic Social Defeat paradigm (CSDS)**

In short, the CD1 aggressor mice were trained and specifically selected on their aggression prior to the start of the experiment. The experimental mice were introduced daily to a novel CD1 resident’s territory, who attacked and forced the experimental mouse into subordination. Defeat sessions lasted until the stress-exposed mouse received three bouts of attacks from the CD1 aggressor or at five minutes in the rare instances when three bouts were not achieved within this duration. Animal health was monitored throughout the experiment to exclude defeat-inflicted injuries. Between daily defeats, stressed mice were housed in the resident’s home cage, but physically separated from the resident by a see-through, perforated mesh barrier, allowing sensory exposure to the CD1 aggressor mouse while preventing further attacks. The defeat time of day was randomized between 11 a.m. and 6 p.m. to avoid habituation and anticipatory behaviors in defeated mice. Non-stressed mice were pair-housed in the same room as the stressed mice with a divider in place to allow sensory but no physical contact. All animals were handled daily and weighed every week. The animals were sacrificed a day after the CSDS ended between 8am and 12am.

**Female Urine Sniffing Test (FUST)**

The males were habituated to a sterile cotton swab applicator inserted into a fresh cage, 1h prior testing. The experiment was carried out in a dimly lit room (LUX 15) and encompassed of 3 phases during which the sniffing behavior was timed. First, 3 minutes exposure to a cotton dip soaked in sterile water. Second, a 45-minute interval without any cotton swab in the cage. Third, 3-minute exposure interval to a cotton tip soaked with female urine. The results are reported as the cumulative time spent sniffing the urine samples.

**Elevated Plus Maze (EPM)**

With the apparatus dimensions matching the published parameters for open arms (30x5x0.5 cm), opposing closed arms (30x5x15 cm) and a center connecting area (5x5x0.5 cm) platform (42). The animals freely explored the maze from for 10 minutes with a constant central starting position facing a closed arm. The maze illumination by ceiling dimmable lights was adjusted to <20 lux in both arms.

1. **Supplemental figures**

**
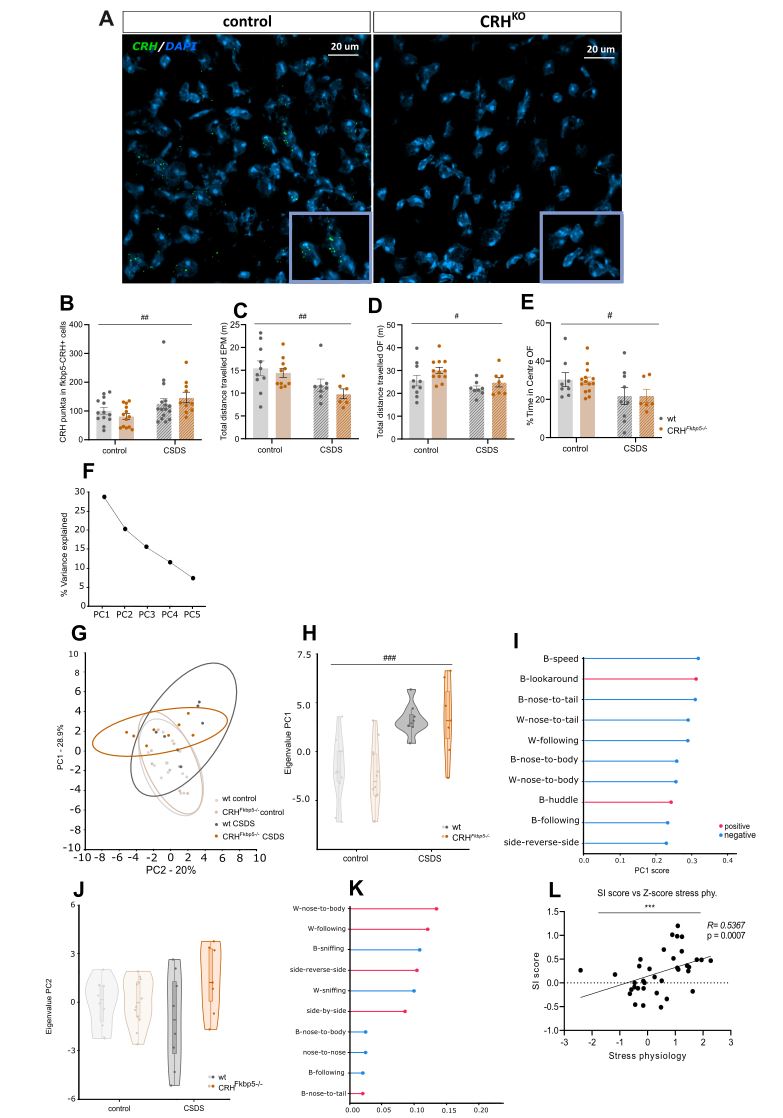
**

**Suppl. Fig. 1:** Verification of CRH RNA scope antibody, CRH puncta quantification and further behavioral analysis of Open Field and Social interaction tasks. (A) RNAscope staining of the CRH probe in CRH-Venus-CD1 line to confirm specificity of our probe. In the KO to the right, no CRH mRNA was detected. (B) Quantification of CRH puncta from the cohort RNAscope quantification of marker expression. Significant upregulation of CRH puncta was counted in the stressed condition (p<0.01), without any genotype effect. (C) Total distance traveled in the elevated plus maze test. Here a stress effect is reported, with the CSDS animals moving significantly less than the controls (p<0.01). (D) Total distance traveled in the open field arena. Here we see again an effect of stressed with the CSDS animals moving significantly less in this setup (*p<0.05*). (E) Quantification of the percentage of time spent out of the 10 minutes in the center of the open field arena. The stressed animals spent significantly less time in the center than the controls (p<0.05). (F) Plotting of the principal components’ contribution to the variance explained in the PCA analysis of the SI. (G) PCA graph of the SI data representing the genotype and stress divided groups. (H) Eigen value violin plot for the PC1 of the PCA analysis, with significant difference between the stressed groups (p<0.001). (I) The top contributing behaviors into the PC1 with positive and negative loading depicted. (J) Violin plot of the Eigen values for PC2 did not reach significant differences between the conditions. (K) The top contributing behaviors to PC2. (L) The Pearson correlation analysis between the SI score of the top contributing behaviors to PC1 and the Stress physiology score.


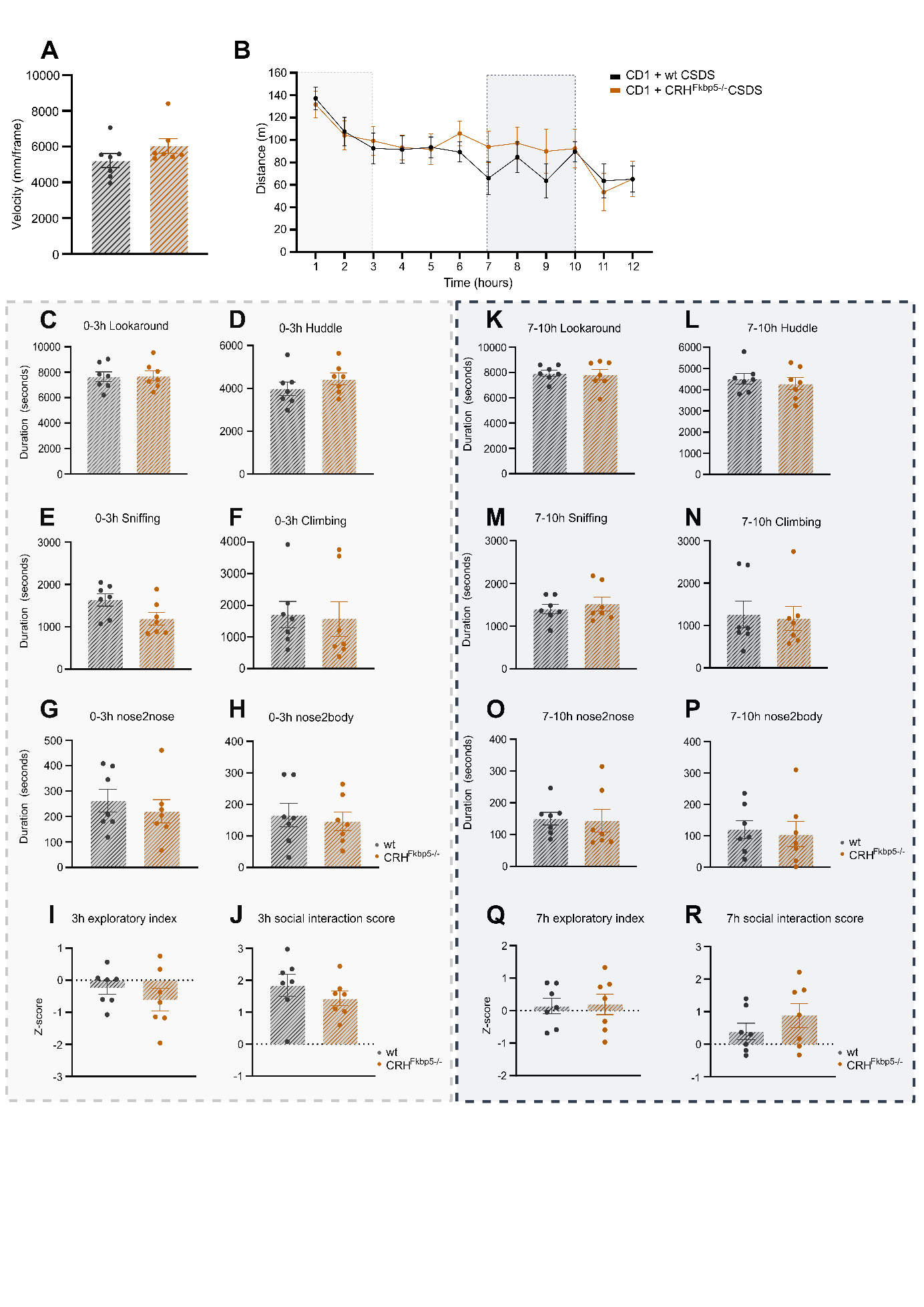


**Suppl Fig. 2:** The behavioral profiling of the CD1 animals in the overnight recordings (A) General velocity of the CD2 animals (B) Line plot of the distance travelled during per hour over the course of the monitoring period. (C)-(H) DeepOF behavioral variables quantified for the 0-3h window. No significant differences between the CD1s behavior towards either wt or CRHFkbp5-/- animals. (I) Exploratory score of the CD1 animals did not differ during the 0-3h period. (J) Social interaction score for 0-3h window of the CD1 did not differ. (K)-(P) The behavioral variables of the CD1s behaviors during the 7-10h window. No significant differences were detected. (Q) The exploratory score of the CD1s during 7-10h time period. (R) The social interaction scores of the CD1s for the time window 7-10h. The scores did not differ between the animals interacting with either wt or CRHFkbp5-/- animals
